# Minimal Residual Disease in Multiple Myeloma: State of the Art and Applications in Clinical Practice

**DOI:** 10.3390/jpm10030120

**Published:** 2020-09-10

**Authors:** Alessandro Gozzetti, Donatella Raspadori, Francesca Bacchiarri, Anna Sicuranza, Paola Pacelli, Ilaria Ferrigno, Dania Tocci, Monica Bocchia

**Affiliations:** Division of Hematology, University of Siena, 53100 Siena, Italy; raspadori@unisi.it (D.R.); francesca.bacchiarri@hotmail.it (F.B.); sicuranza4@unisi.it (A.S.); paolapacelli93@gmail.com (P.P.); ferrigno3@student.unisi.it (I.F.); daniatocci@libero.it (D.T.); bocchia@unisi.it (M.B.)

**Keywords:** multiple myeloma, minimal residual disease, next-generation flow cytometry, next-generation sequencing, complete remission

## Abstract

Novel drugs have revolutionized multiple myeloma therapy in the last 20 years, with median survival that has doubled to up to 8–10 years. The introduction of therapeutic strategies, such as consolidation and maintenance after autologous stem cell transplants, has also ameliorated clinical results. The goal of modern therapies is becoming not only complete remission, but also the deepest possible remission. In this context, the evaluation of minimal residual disease by techniques such as next-generation sequencing (NGS) and next-generation flow (NGF) is becoming part of all new clinical trials that test drug efficacy. This review focuses on minimal residual disease approaches in clinical trials, with particular attention to real-world practices.

## 1. Introduction

Multiple myeloma (MM) is caused by monoclonal plasma cells that proliferate in the bone marrow causing anemia, hypercalcemia, kidney failure, or bone skeletal lesions (i.e., CRAB criteria) [1]. The protein found in the serum or urine of these patients, called the monoclonal protein, is produced by myeloma plasma cells. MM patients presented with a median age above 70 years, and MM incidence has increased in the last 20 years [2], representing 10% in all hematological neoplastic diseases. In the last few years, great improvement has been seen in MM patient survival [1,2,3,4]. This improvement was due to the approval of novel drugs such as proteasome inhibitor (PI) bortezomib (2003) and immunomodulatory drugs (IMIDs) thalidomide and lenalidomide (2006) associated with high-dose therapy represented by autologous stem cell transplant (ASCT) [5,6]. These new drugs associated with novel strategies such as consolidation therapy after transplant and maintenance with lenalidomide (2017) have led to survival prolongation. Extramedullary MM, a peculiar aggressive form of MM related to hematogenous spread of monoclonal plasma cells, also seemed to be improved by the use of novel drugs [7,8,9,10,11]. In the last few years, other drugs have become available, such as second- and third-generation PIs (i.e., Carfilzomib in 2012, Ixazomib in 2018), second-generation IMIDs (Pomalidomide in 2013), and drugs that have new mechanisms of action that were first used in clinical trials and are now commercially available [12], such as the monoclonal antibodies Daratumumab (that targets CD38 on monoclonal plasma cells) and Elotuzumab, which targets “signaling lymphocytic activation molecule F7” (SLAMF-7). Despite this great progress, a small percentage of patients, that is, high-risk cytogenetics patients (10–15%) carrying del 17p and t(4;14), have particularly poor survival and together with patients with other aggressive features (extramedullary disease, renal insufficiency, disease refractoriness, elevated LDH) represent unmet clinical needs [13,14].

### Complete Remission (CR) and Minimal Residual Disease

Novel drugs have improved complete MM responses that are considered survival surrogates. However, it was difficult to deeply define a complete response until a few years ago. In fact, CR was considered when plasma cells were in less than 5% of the bone marrow, and the absence of monoclonal protein was shown in serum or urine by immunofixation or disappearance of soft tissue plasmacytomas. Since most patients relapse even after achieving CR, it became obvious that better techniques are needed to identify minimal residual disease at the lowest possible level. Bone marrow or bone core biopsy analysis can only detect one monoclonal plasma cell out of 100–300 cells with a specificity of 10^−2^. Nowadays, this is not enough considering the depth of response achieved with novel and more efficient drugs. Recent data suggested that more sensitive assays like eight-color next-generation flow (NGF) or next-generation sequencing (NGS) could much further improve minimal residual disease (MRD) detection. International Myeloma Working Group (IMWG) guidelines for response definition identified that a cut-off of 10^−5^ should be considered as the target for the definition of MRD negativity, detected by either NGS or NGF [15,16].

## 2. Next-Generation Flow Cytometry for Evaluation of Multiple Myeloma Minimal Residual Disease

**Figure 1:**
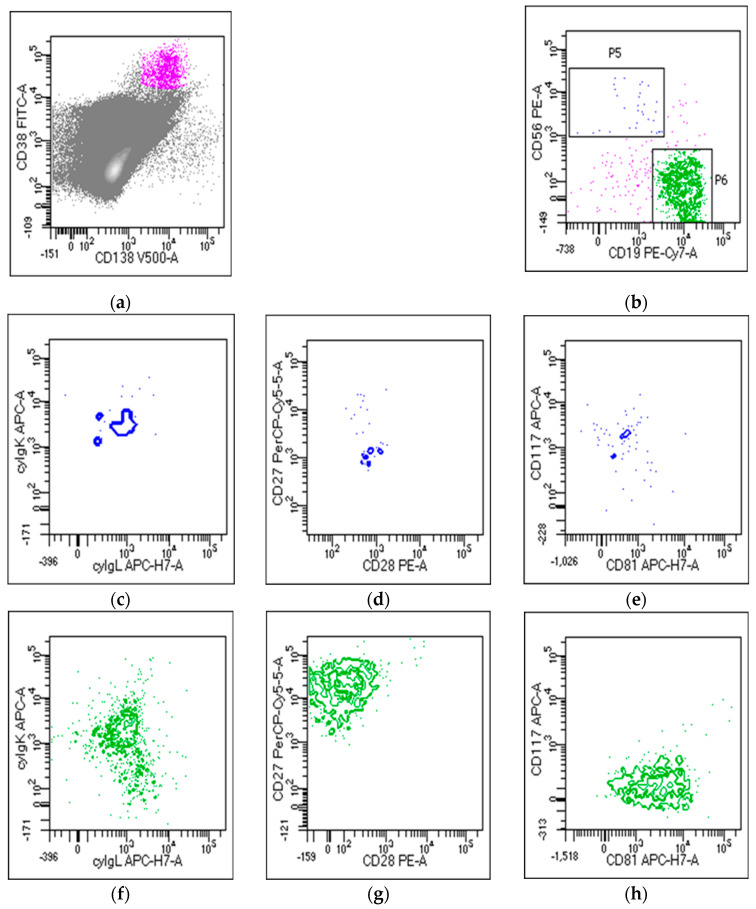
Minimal residual disease (MRD) detection by flow cytometry. (**a**) Plasma cell identification using CD38 and CD138 combination; (**b**) very little amount of abnormal plasma cells expressing CD56 (P5), and large amount of normal plasma cells expressing CD19 (P6) were detected; abnormal plasma cells expressing (**c**) only k light chain, and (**d**) CD28 and (**e**) CD117 antigens; normal plasma cells expressing (**f**) both k and l light chains, and (**g**) CD27 and (**h**) CD81 antigens.

MRD valuation in MM is commonly performed by using two main high-throughput techniques, multiparametric flow cytometry (MFC) and next-generation sequencing (NGS). MFC is widely used in clinical practice to evaluate MM MRD and the depth of complete response (CR) in the bone marrow (BM) of MM patients after therapy [17]. MFC is efficient in detecting and quantifying tumors vs. normal plasma cells (PCs) by using specific markers present on the cell surface or in the cytoplasm. PCs are characterized by the expression of two main markers, CD38 and CD138; however, MM PCs may be recognized because they could express markers such as CD56, CD28, and CD117, and, compared with normal PCs, generally CD45^−low^, CD19^−^, CD27^−^, and CD81^−^. These markers, with the clonal restriction of MM PCs to just one of two immunoglobulin light chains, κ or ʎ, contribute to easily discriminating normal from clonal MM PCs. Older conventional flow cytometric assays are now replaced by advanced assays that permit to simultaneously assess more than eight markers. In fact, EuroFlow Consortium developed NGF, a novel highly sensitive and standardized approach for MM MRD evaluation that is based on the use of two single eight-color tubes containing all needed markers to distinguish normal vs. MM PCs. NGF has many advantages: it is applicable to almost 100% of MM cases, it does not require a diagnostic sample, and it is very fast, requiring just 3–4 h of processing [18]. In this protocol, however, it is necessary to work on fresh samples and acquire a 10^7^ cell/sample to be able to reach the desired and relevant sensitivity levels, expressed as limit of quantification (LOQ) and limit of detection (LOD). The LOQ is calculated as 50 clonal PCs among 10^7^ nucleated cells, and the LOD as 20 clonal PCs among 10^7^ nucleated cells [19]. Thanks to the use of novel high-throughput machines, more sensitive and equipped with more colors to evaluate the expression of a higher number of markers at the same time, NGF today acquires high sensitivity of 10^−5^/10^−6^ that is comparable and superimposable to NGS assays, and the IMWG recommendation is to choose one of the two techniques on the basis of local availability. Despite these advantages, there are still some problems in the use of MFC for MM MRD evaluation. First, we must consider that there are still many differences in the way in which flow cytometry is applied from processing to analytic moment. Some groups prefer to use an in-home antibody mix, many do not determine BM sample quality before processing, and there might be many differences in the way in which data are processed and analyzed, leading to a lack of reproducibility. To address this heterogeneity, the recommendation for laboratories is to harmonize the use of different reagents, fluorochrome panels, sample processing, platforms, and data analysis, and to try to include LOD and LOQ in the final diagnostic report [19]. Another emerging issue was presented with the introduction of anti-CD38 monoclonal antibody therapy daratumumab, which leads to CD38 masking and a limit in PC determination. To address this issue, different methods were developed, such as the introduction of new multiepitope CD38 antibodies that, when added to the mixture of antibodies, permit to evaluate the percentage of MM PCs by binding to CD38 sites that are different from the one occupied by the drug [20]. Other possible solutions to overcome this problem could be to focus on other markers; tests were been done using CD138, whose expression seems to deteriorate over time; CD56, which is not always present on MM PCs; or new markers such as VS38 that, used in place of normal CD38, seems to also have brighter expression in MM PC recognition in patients who started anti-CD38 therapy [21]. An example of MRD evaluation by NGF is given in Figure 1.

## 3. Next-Generation Sequencing (NGS) for Evaluation of Multiple Myeloma MRD

Clonal immunoglobulin (Ig) gene rearrangements represent the main molecular target in MM MRD detection [22]. Allele-specific oligonucleotide polymerase chain reaction (ASO-PCR) was utilized as the first technique to assess MM MRD. However, its applicability is limited by the high rate of somatic hypermutations (SHMs) within Ig heavy chain complementary determinant regions (IgH-CDR). NGS technology, through the parallel sequencing of millions of reads, overcomes technical ASO-PCR pitfalls, allowing for MRD measurement with 10^−6^ sensitivity [23]. The advantage of this approach is represented by the ability to also identify clonality in MM patients with a low tumor burden, offering an improvement in the knowledge of MM biology, providing useful information regarding therapeutic choices and disease management. Moreover, in contrast to ASO-PCR, NGS does not require designing patient-specific primers and a standard curve for “MRD quantification”. Obtained data need to be elaborated through specific bioinformatic tools designed to analyze millions of reads. Two identical sequencing reads were defined as clonotypes, and the >5% frequency of a clonotype, established as the cut-off value for MRD evaluation in follow-up samples, is referred to as clonality [24].

During the last few years, several NGS platforms for MRD detection in MM were tested. Two of these, ImmunoSEQ (Adaptive Biotechnologies, Seattle, WA, USA) and LymphoSIGHT^®^ (Adaptive Biotechnologies, Seattle, WA, USA) both from Adaptive Biotechnologies; the latter, currently known as ClonoSEQ^®^ (Adaptive Biotechnologies, Seattle, WA, USA), was the first licensed by the FDA in 2019, and it is currently the most frequently adopted. These commercial kits are characterized by two-stage and single-reaction PCR amplifying IgH VDJ rearrangements, respectively. Additionally, the LymphoTrack-MiSeq platform was compared to ASO-PCR, as documented by Yao and colleagues; this NGS technology allowed the quantification of those cases defined as positive but not quantifiable (PNQ) by ASO, and it was consequently suggested for MM MRD monitoring [24]. Despite the high sensitivity of NGS, the feasibility of this approach is limited by high costs, long turnaround time, and required expertise. For these reasons, more affordable techniques, such as digital PCR (dPCR), were proposed to be used in daily MM patient management. dPCR does not require a standard curve, leading to the absolute quantification of the target gene, and it is also less laborious than NGS regarding data interpretation. On the other hand, its applicability is not yet standardized, and further studies are needed to confirm it [25,26,27]. Moreover, sample quality is another critical aspect: the hemodilution and patchy nature of MM BM samples may interfere in molecular MRD evaluation, leading to possible false-negative results. Several clinical trials investigated the prognostic value of NGS technology in MM management. Obtained results by a French group (2009 IFM study) on MM patients treated with lenalidomide, bortezomib, and dexamethasone (RVD) showed higher sensitivity for NGS compared with MFC in MRD detection, with MRD negativity established at <10^−6^ [28]. The CASSIOPEIA trial demonstrated that NGS and NGF are superimposable techniques for MRD evaluation. In the PETHEMA study, MRD analysis by deep sequencing at distinct levels was able to identify three MM groups with different time to tumor progression (TTP) [29,30]. In conclusion, on the basis of this evidence, the NGS approach was demonstrated to be a powerful tool for MRD detection, considering the key role of the achievement of MRD negativity in the clinical management of MM patients. In this scenario, the most appealing prospective in MM patients is the use in the clinical practice of this reliable and sensitive technique for MRD assessment. The strong deep-sequencing prognostic role by NGS could lead clinicians to better treatment strategies.

### 3.1. Quality of Bone Marrow Aspirates May Influence MRD Analysis

Both NGF and NGS require high amounts of standardized starting material and sample preparation protocols to optimize the procedure across laboratories. Performing a count of the starting materials, before processing the sample, could help to estimate the number of cells at disposition; however, it is not possible to predict through cell numbers the level of sensitivity we could acquire by NGF, neither is possible to estimate the quality and quantity of DNA/RNA that could be extracted from the sample. Low-quality BM aspirates should be interpreted with caution, especially if they are to be used for specific applications such as MRD quantification [31,32,33]. First, a frequently encountered pitfall in MRD evaluation is BM hemodilution by peripheral blood that may lead to important changes in the distribution of cell populations and to underestimation of neoplastic cells percentages. Different methods were recommended to accurately evaluate the degree of hemodilution. These methods were based on an automated lymphocyte count, PB contamination indices that took account of PC percentages, CD34+ cells, and CD10+ neutrophils [34], or numbers of CD16 bright neutrophils [35]. Moreover, in the case of flow cytometric analysis, NGF can also provide the qualitative assessment of patient samples by allowing for analysis of normal B-cell compartments and non-PC BM cells, such as mast cells or RBCs, which can give us an idea of the hemodilution of analyzed BM samples. The other major problem in MM MRD evaluation is the necessity to acquire a high cell number, especially in the case of flow cytometric analysis, where an acquisition of at least 10^7^ cell/sample is suggested [18]. This problem may be overcome by optimizing the preanalytical procedure through red blood cell (bulk) lysis with ammonium chloride, which gives better results than those of previously employed Ficoll stratification. The use of immunomagnetic CD138+ bead enrichment that isolates neoplastic cells may be helpful in molecular biology tests to selectively and independently analyze the bad quality of BM aspirates; moreover, flow-activated cell sorting (FACS) is preferable, especially in the case of low-infiltrated BM samples, because it provides better recovery of both normal and neoplastic plasma cells [36]. However, we must consider that these two methods could somehow introduce manipulation of BM cells causing problems in MM MRD quantification. Working on high-quality BM samples for MRD evaluation could ameliorate assays, and it is crucial to have good coordination between clinicians and laboratories to improve the accuracy, sensitivity, and specificity of MM MRD detection in MM patients.

### 3.2. MRD in Peripheral Blood

Some studies are exploring the option of alternatively detecting MRD in MM PB samples, overcoming the invasive BM biopsy procedure and with the possibility to detect myeloma plasma cells in peculiar situations such as extramedullary localization. Another reason to search for MRD in PB is to avoid false-negative results due to the patchy distribution of myeloma cells in the bone marrow. NGS, NGF, or analysis of circulating cell-free tumor DNA (ctDNA) in liquid biopsy, in combination with imaging methods for MM MRD monitoring [37], could represent a valid strategy. However, all these efforts did not show so far a clear advantage for PB analysis with respect to BM. In fact, a recent study [38] showed that in 137 naïve MM patients, after treatment 55 (40%) still had detectable disease with NGF in the BM, while MRD was not detectable in PB. NGS in another study was utilized to detect myeloma cells in peripheral blood of 27 MM patients, and there was no difference in responding patients vs. nonresponders [39]. In a French study, ctDNA in PB was negative in 18/26 MM patients that still had disease in BM [40]. At the moment, PB MRD needs to be further investigated in trials that compare BM and PB techniques.

## 4. MRD in Clinical Trials

Despite the most aggressive therapies and the most recent innovative drugs, it has not been possible to predict with certainty long-surviving patients with respect to patients doing poorly. Recently, evidence-based studies focusing on MRD in MM led to redefining response categories, including MRD evaluation, in these categories [16]. Nowadays, almost all MM patients treated at diagnosis with novel drug combinations can achieve a response, and two-thirds can also obtain optimal long-lasting response, that is, >VGPR [41]. Moreover, long-surviving MM patients at >10 years after ASCT and novel drugs are more frequently seen [42,43,44,45]. However, PFS should not be the primary endpoint in future MM clinical trials, since long follow-ups need to have powerful data, and new drug trials can be discouraged by the long time for approval [46,47]. In the past few years, several studies evaluated MRD; indeed, the depth of response was directly correlated with survival improvement [48,49,50,51,52,53]. MRD applications are several in MM [54,55,56,57] and are believed to be some of the most powerful prognosticators [58]. MRD is tested to determine the depth of response, but it may also be important in future treatment decisions in clinical practice, such as continuing or stopping therapy on the basis of positivity or negativity, respectively [58].

In the large French study IFM2009 [59], 700 newly diagnosed MM patients were randomized after three induction cycles with bortezomib, lenalidomide, and dexamethasone (RVD) to autologous transplantation or RVD as consolidation and lenalidomide therapy as maintenance. In total, 289 patients were evaluated by NGS and 475 by multiparametric flow cytometry (MFC). In 92% of the tested patients, it was possible to measure MRD by NGS: among these, MRD-negative patients showed significantly longer PFS than that of MRD-positive patients (3 year PFS, 87% vs. 42%). However, due to the different sensitivity levels of NGS (10^−6^) and MFC (10^−5^), a direct comparison of the two methods was not possible.

The GEM2005 trial [60] compared induction with VMP/VTP in elderly naive patients, and demonstrated that a CR at the immunophenotypic level in the VMP arm was correlated with a longer OS, that is, at 8 years, 66% of the patients in CR by flow analysis were alive. In particular, 22% of the patients (*N* = 34) were in flow CR and had statistically significant longer PFS, with a median of 59 months compared with the 29 months of patients not in CR. Median OS was also not reached, with 55% at 8 years vs. 57 months, respectively. Interestingly, patients in flow CR after VMP achieved a longer response, with PFS (53%) and OS (66%) not being reached at eight years, and this result was also better than the one obtained in patients in flow CR after VTP.

Martinez-Sanchez et al. reported on MRD testing in 53 MM patients treated in the context of the GEM2000 clinical study treated with a multichemotherapy scheme followed by ASCT [61]. MRD was evaluated both with a flow-cytometry technique and with PCR. Patients with PCR-negative MRD showed significantly higher PFS than that of MRD-positive patients (PFS, 68% vs. 28%). Furthermore, in 28 CR patients, 19 had MRD-negative results, while 8 were MRD-positive; the authors observed significantly lower PFS in MRD-positive patients (25%) than that in MRD-negative patients (79%). The Spanish GEM/PETHEMA group reported on 130 MM patients who had achieved at least VGPR at the end of the induction therapy; MRD-negative patients had significantly higher PFS and OS than those of MRD-positive patients. This result was confirmed by both MFC and fluorescent PCR, and both methods showed feasibility in more than 90% of evaluated patients [62]. Several studies proved that the use of triplets and novel therapies like anti-CD38 monoclonal antibody daratumumab positively influence PFS and MRD. In fact, daratumumab was used in the CASTOR study with bortezomib and dexamethasone (D-Vd), and results showed great advantage in MM patients with relapsed refractory disease compared with Vd. This advantage was seen concerning PFS, with cumulative 69% reduction compared with with Vd. Moreover, complete responses were ameliorated after 12 months of therapy (28.8%) with respect to initial analysis (19.2%) [52]. D-Vd led to responses that were fourfold deeper with respect to Vd, with MRD sensitivity of 10^−5^ and 10^−6^. A follow-up evaluating OS is ongoing [63].

Daratumumab plus lenalidomide/dexamethasone (DRd) significantly prolonged PFS compared with lenalidomide/dexamethasone in the POLLUX study (median PFS not reached vs. 17.5 months, respectively). Moreover, objective response rate (ORR) was 92.9% vs. 76.4%, CR 51% vs. 21%. MRD was also different, 26% vs. 6%, calculated at 10^−5^ [64]. The CASSIOPEIA study confirmed there was a benefit of daratumumab in achieving MRD negativity after consolidation, with increased PFS for D-VTd- vs. VTd-treated patients [65]. In particular, only patients who had achieved complete response or better showed a significant advantage of adding daratumumab to VTd. Ongoing analyses are exploring the relationship between MRD and M-protein clearance [66]. Another study, MAIA, explored the use of daratumumab plus lenalidomide and dexamethasone vs. lenalidomide plus dexamethasone in newly diagnosed patients (DRd vs. Rd); >CR patients were doubled with DRd, and patients who were MRD-negative had more than 3 times as high DRd vs. Rd [67]. Particularly, CR or better was present in 47.6% vs. 24.9% in DRd vs. Rd, respectively. Of the DRd group, 24% of patients were MRD-negative vs. 7% in the control group (MRD calculated at 10^−5^), and these data were statistically significant.

Another study showed the efficacy of the carfilzomib–lenalidomide–dexamethasone (KRd) regimen in untreated multiple myeloma patients concerning depth of response [68]. KRd could give a durable and deep response. MRD negativity was calculated in this study with at least 10^−5^ sensitivity [69]. This work confirmed that patients with MRD-negative CR had better PFS and about 80% less disease progression than those of the control group.

Flow-measured MRD was a primary endpoint in a study in which MM patients were treated with lenalidomide, Velcade, and dexamethasone (RVd) at diagnosis, followed by autologous stem cell transplantation and R maintenance [70]. ORR was 89% with 53% flow MRD-negative patients, half of which were negative by PCR. As of this moment, 36% (*n* = 29) of patients are continuing therapy with R, and 66% of them maintained MRD negativity at a median follow-up of 27 months. In this study, MRD negativity was shown to be a prognostic factor for PFS. The IFM showed that ASCT associated with RVd as initial treatment plus consolidation, and R maintenance was able to obtain very good and deep responses with a good safety profile in untreated MM patients. In particular, 58% of patients achieved a CR, with 68% flow MRD negativity [71]. MRD was also tested in the setting of consolidation after transplant. In the recent randomized multicenter study EMN02/HO95, advantage in PFS for ASCT compared with VMP was shown in untreated MM patients and for RVD as consolidation therapy compared with observation [72]. In this study, MRD was detected by flow, and this showed that patients with negative status before maintenance had longer PFS vs. that of MRD-positive patients. Preliminary data published by our group also seemed in favor of the use of MRD to monitor therapy efficacy: in particular, the use of daratumumab as consolidation therapy after VTD-based induction and autologous transplant could efficiently improve MRD status [73].

Lastly, in a recent meta-analysis, MRD was correlated with PFS in MM patients treated at diagnosis [45]. The study examined six randomized trials in which MRD status was evaluated after therapy (by NGF, NGS, and multiparametric flow), and correlated data with obtained responses and PFS. In all studies, MRD negativity was directly related to improved survival, and PFS in particular.

Previously, two other studies analyzed MRD status in MM patients at first treatment, and confirmed that MRD negativity is correlated with prolonged survival. Meta-analysis was done on four previous studies, and found statistically significant difference regarding PFS and OS between patients who had achieved MRD negativity and those who had not [74]. Other analyzed data from 14 studies confirmed MRD negativity with statistical significance as surrogate for PFS and OS [70].

MRD detection in the bone marrow should be accompanied by skeletal evaluation. Several studies showed that the detection of positive lesions in FDG-PET/CT, both at diagnosis and recurrence, has important prognostic significance [75,76]. Studies reporting NGF and NGS are summarized in Table 1. 

### 4.1. MRD in High-Risk Cytogenetic MM Patients

Patients with high-risk features, that is, del 17p, t 4;14, and 14;16, may benefit from an informative strategy about MRD. These patients can achieve CR after first treatment, but they usually experience early relapse. Although not yet clinically recommended, it may be good to detect early MRD relapse information on periodic follow-ups to eventually switch the therapy. In the CASSIOPEA study, a benefit was observed concerning PFS and number of MRD-negative MM patients, that is, high-risk cytogenetic profile and ISS III [65]. Recently, Li et al. [77] demonstrated that those patients that had achieved MRD negativity had better PFS and OS. This was true for standard- and high-risk patients (NR vs. 26 months, respectively). However, independently of cytogenetics, all patients (*n* = 123) had better survival if MRD-negative (*n* = 31) when compare with those who were MRD-positive (*n* = 92; NR vs. 26 months).

### 4.2. Imaging in MRD Evaluation

Imaging is very important for MRD assessment in MM. Since bone disease is the most frequent presentation of the disease, monoclonal plasma cells can persist after therapy as focal lesions or as diffuse infiltration in the bone representing an incomplete response to therapy. This residual disease could be also cause of relapse in MM patients. With this in mind, it is recommended by the IMWG criteria to evaluate imaging after therapy to assess response [78]. FDG-PET is a functional imaging technique that detects bone disease in MM and it is optimal to assess MRD [78]. The tracer used in this imaging is a glucose analog (FDG) that permits to detect active disease.

In an Italian study conducted on diagnosed myeloma patients undergoing a transplant procedure, the persistence of PET/TC positivity after induction therapy was predictive of early recurrence; in contrast, post-transplant negative PET/CT (Day +100) was associated with statistically significant longer PFS and OS with respect to patients with positive PET-CT [79]. The authors showed that the survival of CR PET/CT-positive patients was significantly lower than that of CR PET/CT-negative patients, that is, PFS 44 vs. 84 months, and five-year OS 70% vs. 90%, respectively. Similar results were reported in the IFM2009 study; the PFS of patients in whom PET/CT normalization occurred at the end of induction therapy and before maintenance therapy was much better than those of PET/CT-positive patients [80].

## 5. MRD in Real-World Clinical Practice: More Questions than Answers

Nowadays, it is not recommended to make therapeutic decisions on the basis of MRD status in clinical practice. However, many trials are now exploring consolidation or maintenance therapy based on MRD. It is now obvious that MRD is a surrogate for PFS and ultimately OS. It is also exciting to see that MRD could be a surrogate for novel drugs to be approved for MM. The utility of the technique is undeniable. However, many questions arise in clinical practice: is a single MRD evaluation enough at one timepoint? Are 3 months after ASCT and 1 month after nontransplant therapy optimal timepoints for first evaluation? Should MRD be searched at 1 year or later after therapy or during maintenance to show sustained MRD negativity? Sustained MRD negativity (at 1 or 2 year(s) after therapy) is the goal to predict prolonged PFS. Either NGF or NGS associated with imaging PET/CT are being used in some hematological centers, even outside clinical trials, to achieve personal experience. Data coming from single-center studies showed that MM patients in sustained CR at later than 2 years were mostly MRD-negative, or could regress to a monoclonal gammopathy of undetermined significance (MGUS)-like disease with a high percentage of polyclonal vs. clonal plasma cells, indicating that a small MM clone could survive but also be formally contained [81]. MRD detection by NGS ClonoSeq has been the first approved by FDA, and many centers are now utilizing an external service for diagnostic purpose. Implementing in-house laboratory methodologies will be certainly convenient in terms of financial advantage.

## 6. Conclusions

Both NGF and NGS are available techniques recognized by the IMWG for the detection of MRD. In-house availability and laboratory experience drive the choice between the two at the moment. Although MRD detection is not recommended for clinical therapeutic decisions, real-world practice can be very informative to test depth and duration of response in patients treated with novel drugs or autotrasplant. This is an exciting era for MM management. We use the word “incurable” for many diseases, and “MM” is one of these. However, many drugs are available with different mechanisms of action that hold a premise for a bright future: “cure” is finally sometimes mentioned, which can be achieved with better disease knowledge, disease prognosis, therapies, and methodologies to detect the disease at the deepest possible level.

## Figures and Tables

**Table 1:** Next-generation flow (NGF) and next-generation sequencing (NGS) studies on reported MRD in the literature. The symbol * indicate the studies in the literature.

Author	NGF			NGS	
	1 × 10^−4^	1 × 10^−5^	1 × 10^−6^	1 × 10^−5^	1 × 10^−6^
Paiva et al. [17]		*	*		*
Flores-Montero et al. [18]		*	*		
Oliva et al. [30]		*			*
Rawstron et al. [21]	*				
Bai et al. [23]			*		*
Yao et al. [24]		*		*	*
Perrot et al. [27]					*
Avet-Loiseau et al. [28]					*
Martinez-Lopez et al. [29]				*	

## References

[1] Gozzetti A., Candi V., Papini G., Bocchia M. (2014). Therapeutic advancements in multiple myeloma. Front. Oncol..

[2] Howlader N., Noone A.M., Krapcho M., Miller D., Brest A., Yu M., Ruhl J., Tatalovich Z., Mariotto A., Lewis D.R. (2020). SEER Cancer Statistics Review, 1975–2017.

[3] Mohty M., Terpos E., Mateos M.V., Cavo M., Lejniece S., Beksac M., Bekadja M.A., Legiec W., Dimopoulos M., Stankovic S. (2018). EMMOS Investigators. Multiple Myeloma Treatment in Real-world Clinical Practice: Results of a Prospective, Multinational, Noninterventional Study. Clin. Lymphoma Myeloma Leuk..

[4] Brenner H., Gondos A., Pulte D. (2008). Recent major improvement in long-term survival of younger patients with multiple myeloma. Blood.

[5] Cini M., Zamagni E., Valdré L., Palareti G., Patriarca F., Tacchetti P., Legnani C., Catalano L., Masini L., Tosi P. (2010). Thalidomide–dexamethasone as upfront therapy for patients with newly diagnosed multiple myeloma: Thrombophilic alterations, thrombotic complications, and thromboprophylaxis with low-dose warfarin. Eur. J. Haematol..

[6] Kumar S.K., Rajkumar S.V., Dispenzieri A., Lacy M.Q., Hayman S.R., Buadi F.K., Zeldenrust S.R., Dingli D., Russell S.J., Lust J.A. (2008). Improved survival in multiple myeloma and the impact of novel therapies. Blood.

[7] Gozzetti A., Cerase A., Lotti F., Rossi D., Palumbo A., Petrucci M.T., Patriarca F., Nozzoli C., Cavo M., Offidani M. (2012). GIMEMA (Gruppo Italiano Malattie Ematologiche dell’Adulto) Myeloma Working Party Extramedullary intracranial localizations of multiple myeloma and treatment with novel agents: A retrospective survey of 50 patients. Cancer.

[8] Jurczyszyn A., Grzasko N., Gozzetti A., Czepiel J., Cerase A., Hungria V., Crusoe E., Silva Dias A.L., Vij R., Fiala M.A. (2016). Central nervous system involvement by multiple myeloma: A multi-institutional retrospective study of 172 patients in daily clinical practice. Am. J. Hematol..

[9] Gozzetti A., Cerase A. (2014). Novel agents in CNS myeloma treatment. Cent. Nerv. Syst. Agents Med. Chem..

[10] Castillo J.J., Jurczyszyn A., Brozova L., Crusoe E., Czepiel J., Davila J., Dispenzieri A., Eveillard M., Fiala M.A., Ghobrial I.M. (2017). IgM myeloma: A multicenter retrospective study of 134 patients. Am. J. Hematol..

[11] Jurczyszyn A., Olszewska-Szopa M., Hungria V., Crusoe E., Pika T., Delforge M., Leleu X., Rasche L., Nooka A.K., Druzd-Sitek A. (2016). Cutaneous involvement in multiple myeloma: A multi-institutional retrospective study of 53 patients. Leuk. Lymphoma.

[12] Ocio E.M., Richardson P.G., Rajkumar S.V., Palumbo A., Mateos M.V., Orlowski R., Kumar S., Usmani S., Roodman D., Niesvizky R. (2014). New drugs and novel mechanisms of action in multiple myeloma 2013: A report from the International Myeloma Working group (IMWG). Leukemia.

[13] Rajan A.M., Rajkumar S.V. (2015). Interpretation of cytogenetic results in multiple myeloma for clinical practice. Blood Cancer J..

[14] Gozzetti A., Le Beau M.M. (2000). Fluorescence in situ hybridization: Uses and limitations. Semin Hematol..

[15] Landgren O., Rajkumar S.V. (2016). New Developments in Diagnosis, Prognosis, and Assessment of Response in Multiple Myeloma. Clin. Cancer Res..

[16] Kumar S., Paiva B., Anderson K.C., Durie B., Landgren O., Moreau P., Munshi N., Lonial S., Bladé J., Mateos M.V. (2016). International Myeloma Working Group consensus criteria for response and minimal residual disease assessment in multiple myeloma. Lancet Oncol..

[17] Paiva B., Gutierrez N.C., Rosinol L., Vidriales M.B., Montalban M.A., Martinez-Lopez J., Mateos M.V., Cibeira M.T., Cordon L., Oriol A. (2012). High-risk cytogenetics and persistent minimal residual disease by multiparametric flow cytometry predict unsustained complete response after autologous stem cell transplantation in multiple myeloma. Blood.

[18] Flores-Montero J., Sanoja-Flores L., Paiva B., Puig N., García-Sánchez O., Böttcher S., van der Velden V., Pérez-Morán J.J., Vidriales M.B., García-Sanz R. (2017). Next Generation Flow for highly sensitive and standardized detection of minimal residual disease in multiple myeloma. Leukemia.

[19] Arroz M., Came N., Lin P., Chen W., Yuan C., Lagoo A., Monreal M., De Tute R., Vergilio J.A., Rawstron A.C. (2016). Consensus guidelines on plasma cell myeloma minimal residual disease analysis and reporting. Cytom. B Clin. Cytom..

[20] Oberle A., Brandt A., Alawi M., Langebrake C., Janjetovic S., Wolschke C., Schütze K., Bannas P., Kröger N., Koch-Nolte F. (2017). Long-term CD38 saturation by daratumumab interferes with diagnostic myeloma cell detection. Haematologica.

[21] Courville E.L., Yohe S., Shivers P., Linden M.A. (2020). VS38 Identifies Myeloma Cells With Dim CD38 Expression and Plasma Cells Following Daratumumab Therapy, Which Interferes With CD38 Detection for 4 to 6 Months. Am. J. Clin. Pathol..

[22] Lionetti M., Neri A. (2017). Utilizing next-generation sequencing in the management of multiple myeloma. Expert Rev. Mol. Diagn..

[23] Bai Y., Orfao A., Chim C.S. (2018). Molecular Detection of Minimal Residual Disease in Multiple Myeloma. Br. J. Haematol..

[24] Yao Q., Bai Y., Orfao A., Chim C.S. (2019). Standardized Minimal Residual Disease Detection by Next-Generation Sequencing in Multiple Myeloma. Front. Oncol..

[25] Della Starza I., Nunes V., Cavalli M., De Novi L.A., Ilari C., Apicella V., Vitale A., Testa A.M., Del Giudice I., Chiaretti S. (2016). Comparative Analysis Between RQ-PCR and digital-droplet-PCR of immunoglobulin/T-cell Receptor Gene Rearrangements to Monitor Minimal Residual Disease in Acute Lymphoblastic Leukaemia. Br. J. Haematol..

[26] Korde N., Mailankody S., Roschewski M., Faham M., Kotwaliwale C., Moorhead M., Kwok M.L., Manasanch E.E., Bhutani M., Tageja N. (2014). Minimal Residual Disease (MRD) Testing in Newly Diagnosed Multiple myeloma (MM) Patients: A Prospective Head-to-Head Assessment of Cell-Based, Molecular, and Molecular-Imaging Modalities. Blood.

[27] Perrot A., Lauwers-Cances V., Corre J., Robillard N., Hulin C., Chretien M.L., Dejoie T., Maheo S., Stoppa A.M., Pegourie B. (2018). Minimal residual disease negativity using deep sequencing is a major prognostic factor in multiple myeloma. Blood.

[28] Avet-Loiseau H., Bene M.C., Wuilleme S., Corre J., Attal M., Arnulf B., Garderet L., Macro M., Stoppa A.M., Delforge M. (2019). Concordance of Post-consolidation Minimal Residual Disease Rates by Multiparametric Flow Cytometry and Next-generation Sequencing in CASSIOPEIA. Clin. Lymphoma Myeloma Leuk..

[29] Martinez-Lopez J., Lahuerta J.J., Pepin F., Gonzalez M., Barrio S., Alaya R., Puig N., Montalban M.A., Paiva B., Weng L. (2014). Prognostic value of deep sequencing method for minimal residual disease detection in multiple myeloma. Blood.

[30] Oliva S., Gambella M., Gilestro M., Muccio V.E., Gay F., Drandi D., Ferrero S., Passera R., Pautasso C., Bernardini A. (2017). Minimal residual disease after transplantation or lenalidomide-based consolidation in myeloma patients: A prospective analysis. Oncotarget.

[31] Rawstron A.C., Orfao A., Beksac M., Bezdickova L., Brooimans R.A., Bumbea H., Dalva K., Fuhler G., Gratama J., Hose D. (2008). European Myeloma Network. Report of the European Myeloma Network on Multiparametric Flow Cytometry in Multiple Myeloma and Related Disorders. Haematologica.

[32] Brooimans R.A., Kraan J., Van Putten W., Cornelissen J.J., Löwenberg B., Gratama J.W. (2009). Flow cytometric differential of leukocytes populations in normal bone marrow: Influence of peripheral blood contamination. Cytom. B Clin. Cytom..

[33] Gupta R., Bhaskar A., Kumar L., Sharma A., Jain P. (2009). Flow Cytometric Immunophenotyping and Minimal Residual Disease Analysis in Multiple Myeloma. Am. J. Clin. Pathol..

[34] Delgado J.A., Guillén-Grima F., Moreno C., Panizo C., Pérez-Robles C., Mata J.J., Moreno L., Arana P., Chocarro S., Merino J. (2017). A simple flow-cytometry method to evaluate peripheral blood contamination of bone marrow aspirates. J. Immunol. Methods.

[35] Loken M.R., Chu S.C., Fritschle W., Kalnoski M., Wells D.A. (2008). Normalization of bone marrow aspirates for hemodilution in flow cytometric analyses. Cytom. B Clin. Cytom..

[36] Paiva B., Puig N., Cedena M.T., De Jong B.G., Ruiz Y., Rapado I., Martinez-Lopez J., Cordon L., D’Alignani D., Delgado J.A. (2017). Differentiation stage of myeloma plasma cells: Biological and clinical significance. Leukemia.

[37] Sanoja-Flores L., Flores-Montero J., Puig N., Contreras-Sanfeliciano T., Pontes R., Corral-Mateos A., García-Sánchez O., Díez-Campelo M., de Pessoa Magalhães R.J., García-Martín L. (2019). Blood monitoring of circulating tumor plasma cells by next generation flow in multiple myeloma after therapy. Blood.

[38] Oberle A., Brandt A., Voigtlaender M., Thiele B., Radloff J., Schulenkorf A., Alawi M., Akyüz N., März M., Ford C.T. (2017). Monitoring multiple myeloma by next-generation sequencing of V(D)J rearrangements from circulating myeloma cells and cell-free myeloma DNA. Haematologica.

[39] Biancon G., Gimondi S., Vendramin A., Carniti C., Corradini P. (2018). Noninvasive molecular monitoring in multiple myeloma patients using cell-free tumor DNA: A pilot study. J. Mol. Diagn..

[40] Mazzotti C., Buisson L., Maheo S., Perrot A., Chretien M.L., Leleu X., Hulin C., Manier S., Hébraud B., Roussel M. (2018). Myeloma MRD by deep sequencing from circulating tumor DNA does not correlate with results obtained in the bone marrow. Blood Adv..

[41] Landgren O., Owen R.G. (2016). Better therapy requires better response evaluation: Paving the way for minimal residual disease testing for every myeloma patient. Cytom. B Clin. Cytom..

[42] Durie B.G., Hoering A., Abidi M.H., Rajkumar S.V., Epstein J., Kahanic S.P., Thakuri M., Reu F., Reynolds C.M., Sexton R. (2017). Bortezomib with lenalidomide and dexamethasone versus lenalidomide and dexamethasone alone in patients with newly diagnosed myeloma without intent for immediate autologous stem–cell transplant (SWOG S0777): A randomised, open–label, phase 3 trial. Lancet.

[43] Attal M., Lauwers-Cances V., Marit G., Caillot D., Moreau P., Facon T., Stoppa A.M., Hulin C., Benboubker L., Garderet L. (2012). Lenalidomide maintenance after stem-cell transplantation for multiple myeloma. N. Engl. J. Med..

[44] McCarthy P.L., Owzar K., Hofmeister C.C., Hurd D.D., Hassoun H., Richardson P.G., Giralt S., Stadtmauer E.A., Weisdorf D.J., Vij R. (2012). Lenalidomide after stem-cell transplantation for multiple myeloma. N. Engl. J. Med..

[45] Avet-Loiseau H., Ludwig H., Landgren O., Paiva B., Morris C., Yang H., Zhou K., Ro S., Mateos M.V. (2020). Minimal Residual Disease Status as a Surrogate Endpoint for Progression-free Survival in Newly Diagnosed Multiple Myeloma Studies: A Meta-analysis. Clin. Lymphoma Myeloma Leuk..

[46] Gandolfi S., Prada C.P., Richardson P.G. (2018). How I treat the young patient with multiple myeloma. Blood.

[47] Touzeau C., Moreau P., Dumontet C. (2017). Monoclonal antibody therapy in multiple myeloma. Leukemia.

[48] Paiva B., Vidriales M.B., Cervero J., Mateo G., Pérez J.J., Montalbán M.A., Sureda A., Montejano L., Gutiérrez N.C., de García Coca A. (2008). Multiparameter flow cytometric remission is the most relevant prognostic factor for multiple myeloma patients who undergo autologous stem cell transplantation. Blood.

[49] Korde N., Roschewski M., Zingone A., Kwok M., Manasanch E.E., Bhutani M., Tageja N., Kazandjian D., Mailankody S., Wu P. (2015). Treatment with carfilzomib–lenalidomide–dexamethasone with lenalidomide extension in patients with smoldering or newly diagnosed multiple myeloma. JAMA Oncol..

[50] Lonial S., Anderson K.C. (2014). Association of response endpoints with survival outcomes in multiple myeloma. Leukemia.

[51] Lonial S., Dimopoulos M., Palumbo A., White D., Grosicki S., Spicka I., Walter-Croneck A., Moreau P., Mateos M.V., Magen H. (2015). ELOQUENT-2 Investigators. Elotuzumab therapy for relapsed or refractory multiple myeloma. N. Engl. J. Med..

[52] Palumbo A., Chanan-Khan A., Weisel K., Nooka A.K., Masszi T., Beksac M., Spicka I., Hungria V., Munder M., Mateos M.V. (2016). CASTOR Investigators. Daratumumab, bortezomib, and dexamethasone for multiple myeloma. N. Engl. J. Med..

[53] Stewart A.K., Rajkumar S.V., Dimopoulos M.A., Masszi T., Špička I., Oriol A., Hájek R., Rosiñol L., Siegel D.S., Mihaylov G.G. (2015). Carfilzomib, lenalidomide, and dexamethasone for relapsed multiple myeloma. N. Engl. J. Med..

[54] Munshi N., Anderson K.C. (2013). Minimal residual disease in multiple myeloma. J. Clin. Oncol..

[55] Mailankody S., Korde N., Lesokhin A.M., Lendvai N., Hassoun H., Stetler-Stevenson M., Landgren O. (2015). Minimal residual disease in multiple myeloma: Bringing the bench to the bedside. Nat. Rev. Clin. Oncol..

[56] Rawstron A.C., de Tute R.M., Haughton J., Owen R.G. (2016). Measuring disease levels in myeloma using flow cytometry in combination with other laboratory techniques: Lessons from the past 20 years at the Leeds Haematological Malignancy Diagnostic Service. Cytom. B Clin. Cytom..

[57] Rawstron A.C., Child J.A., de Tute R.M., Davies F.E., Gregory W.M., Bell S.E., Szubert A.J., Navarro-Coy N., Drayson M.T., Feyler S. (2013). Minimal residual disease assessed by multiparameter flow cytometry in multiple myeloma: Impact on outcome in the Medical Research Council Myeloma IX Study. J. Clin. Oncol..

[58] Rajkumar S.V., Harousseau J.L., Durie B., Anderson K.C., Dimopoulos M., Kyle R., Blade J., Richardson P., Orlowski R., Siegel D. (2011). Consensus recommendations for the uniform reporting of clinical trials: Report of the International Myeloma Workshop Consensus Panel. Blood.

[59] Attal M., Lauwers-Cances V., Hulin C., Leleu X., Caillot D., Escoffre M., Arnulf B., Macro M., Belhadj K., Garderet L. (2017). IFM 2009 Study. Lenalidomide, Bortezomib, and Dexamethasone with Transplantation for Myeloma. N. Engl. J. Med..

[60] Mateos M.V., Oriol A., López J.M., Teruel A.I., López de la Guía A., López J., Bengoechea E., Pérez M., Martínez R., Palomera L. (2014). GEM2005 Trial Update Comparing VMP/VTP as Induction in Elderly Multiple Myeloma Patients: Do We Still Need Alkylators?. Blood.

[61] Martínez-Sánchez P., Montejano L., Sarasquete M.E., García-Sanz R., Fernández-Redondo E., Ayala R., Montalbán M.A., Martínez R., García Laraña J., Alegre A. (2008). Evaluation of minimal residual disease in multiple myeloma patients by fluorescent-polymerase chain reaction: The prognostic impact of achieving molecular response. Br. J. Haematol..

[62] Martinez-Lopez J., Fernández-Redondo E., García-Sánz R., Montalbán M.A., Martínez-Sánchez P., Pavia B., Mateos M.V., Rosiñol L., Martín M., Ayala R. (2013). Clinical applicability and prognostic significance of molecular response assessed by fluorescent-PCR of immunoglobulin genes in multiple myeloma. Results from a GEM/PETHEMA study. Br. J. Haematol..

[63] Spencer A., Lentzsch S., Weisel K., Avet-Loiseau H., Mark T.M., Spicka I., Masszi T., Lauri B., Levin M.D., Bosi A. (2018). Daratumumab plus bortezomib and dexamethasone versus bortezomib and dexamethasone in relapsed or refractory multiple myeloma: Updated analysis of CASTOR. Haematologica.

[64] Dimopoulos M.A., San-Miguel J., Belch A., White D., Benboubker L., Cook G., Leiba M., Morton J., Ho P.J., Kim K. (2018). Daratumumab plus lenalidomide and dexamethasone versus lenalidomide and dexamethasone in relapsed or refractory multiple myeloma: Updated analysis of POLLUX. Haematologica.

[65] Moreau P., Attal M., Hulin C., Arnulf B., Belhadj K., Benboubker L., Béné M.C., Broijl A., Caillon H., Caillot D. (2019). Bortezomib, thalidomide, and dexamethasone with or without daratumumab before and after autologous stem-cell transplantation for newly diagnosed multiple myeloma (CASSIOPEIA): A randomised, open-label, phase 3 study. Lancet.

[66] Mills J.R., Barnidge D.R., Dispenzieri A., Murray D.L. (2017). High sensitivity blood-based M-protein detection in sCR patients with multiple myeloma. Blood Cancer J..

[67] Facon T., Kumar S., Plesner T., Orlowski R.Z., Moreau P., Bahlis N., Basu S., Nahi H., Hulin C., Quach H. (2019). Daratumumab plus Lenalidomide and Dexamethasone for Untreated Myeloma. N. Engl. J. Med..

[68] Kazandjian D., Korde N., Mailankody S., Hill E., Figg W.D., Roschewski M., Landgren O. (2018). Remission and Progression-Free Survival in Patients with Newly Diagnosed Multiple Myeloma Treated With Carfilzomib, Lenalidomide, and Dexamethasone: Five-Year Follow-up of a Phase 2 Clinical Trial. JAMA Oncol..

[69] Landgren O., Giralt S. (2016). MRD-driven treatment paradigm for newly diagnosed transplant eligible multiple myeloma patients. Bone Marrow Transplant..

[70] Luoma S., Anttila P., Säily M., Lundan T., Heskanen J., Siitonen T., Kakko S., Putkonen M., Ollikainen H., Terävä V. (2019). RVD induction and autologous stem cell transplantation followed by lenalidomide maintenance in newly diagnosed multiple myeloma: A phase 2 study of the Finnish Myeloma Group. Ann. Hematol..

[71] Roussel M., Lauwers-Cances V., Robillard N., Hulin C., Leleu X., Benboubker L., Marit G., Moreau P., Pegourie B., Caillot D. (2014). Front-Line Transplantation Program With Lenalidomide, Bortezomib, and Dexamethasone Combination As Induction and Consolidation Followed by Lenalidomide Maintenance in Patients With Multiple Myeloma: A Phase II Study by the Intergroupe Francophone du Myelome. J. Clin. Oncol..

[72] Cavo M., Gay F., Beksac M., Pantani L., Petrucci M.T., Dimopoulos M.A., Dozza L., van der Holt B., Zweegman S., Oliva S. (2020). Autologous haematopoietic stem-cell transplantation versus bortezomib–melphalan–prednisone, with or without bortezomib–lenalidomide–dexamethasone consolidation therapy, and lenalidomide maintenance for newly diagnosed multiple myeloma (EMN02/HO95): A multicentre, randomised, open-label, phase 3 study. Lancet Haematol..

[73] Gozzetti A., Raspadori D., Bacchiarri F., Pacelli P., Di Martino F., Liberati A.M., Sicuranza A., Lombardo A., Caffarelli C., Antonioli E. (2019). DART4MM: Daratumumab as consolidation therapy in patients who already achieved optimal response/MRD positivity by next generation flow (NGF): Preliminary results of a phase 2 multicenter study. Clin. Lymphoma Myeloma Leuk..

[74] Munshi N.C., Avet-Loiseau H., Rawstron A.C., Owen R.G., Child J.A., Thakurta A., Sherrington P., Samur M.K., Georgieva A., Anderson K.C. (2017). Association of minimal residual disease with superior survival outcomes in patients with multiple myeloma: A metaanalysis. JAMA Oncol..

[75] Usmani S.Z., Mitchell A., Waheed S., Crowley J., Hoering A., Petty N., Brown T., Bartel T., Anaissie E., van Rhee F. (2013). Prognostic implications of serial 18-fluoro-deoxyglucose emission tomography in multiple myeloma treated with total therapy 3. Blood.

[76] Bartel T.B., Haessler J., Brown T.L., Shaughnessy Jr J.D., van Rhee F., Anaissie E., Alpe T., Angtuaco E., Walker R., Epstein J. (2009). F18-fluorodeoxyglucose positron emission tomography in the context of other imaging techniques and prognostic factors in multiple myeloma. Blood.

[77] Li H., Li F., Zhou X., Mei J., Song P., An Z., Zhao Q., Guo X., Wang X., Zhai Y. (2019). Achieving minimal residual disease-negative by multiparameter flow cytometry may ameliorate a poor prognosis in MM patients with high-risk cytogenetics: A retrospective single-center analysis. Ann. Hematol..

[78] Jamet B., Zamagni E., Nanni C., Bailly C., Carlier T., Touzeau C., Michaud A.V., Moreau P., Bodet-Milin C., Kraeber-Bodere F. (2020). Functional Imaging for Therapeutic Assessment and Minimal Residual Disease Detection in Multiple Myeloma. Int. J. Mol. Sci..

[79] Zamagni E., Patriarca F., Nanni C., Zannetti B., Englaro E., Pezzi A., Tacchetti P., Buttignol S., Perrone G., Brioli A. (2011). Prognostic relevance of 18-F FDG PET/CT in newly diagnosed multiple myeloma patients treated with up-front autologous transplantation. Blood.

[80] Moreau P., Attal M., Caillot D., Macro M., Karlin L., Garderet L., Facon T., Benboubker L., Escoffre-Barbe M., Stoppa A.M. (2017). Prospective evaluation of MRI and PET-CT at diagnosis and before maintenance therapy in symptomatic patients with multiple myeloma included in the IFM/DFCI 2009 trial. J. Clin. Oncol..

[81] Terpos E., Kostopoulos I.V., Kastritis E., Ntanasis-Stathopoulos I., Migkou M., Rousakis P., Argyriou A.T., Kanellias N., Fotiou D., Eleutherakis-Papaiakovou E. (2019). Impact of Minimal Residual Disease Detection by Next-Generation Flow Cytometry in Multiple Myeloma Patients with Sustained Complete Remission after Frontline Therapy. HemaSphere.

